# Supplementary figures and images for: Extensive trimming of short single-stranded DNA oligonucleotides during replication-coupled gene editing in mammalian cells
Source: PLoS Genet. 2020 Oct 29;16(10):e1009041. doi: 10.1371/journal.pgen.1009041 (PMC7595315; doi:10.1371/journal.pgen.1009041)

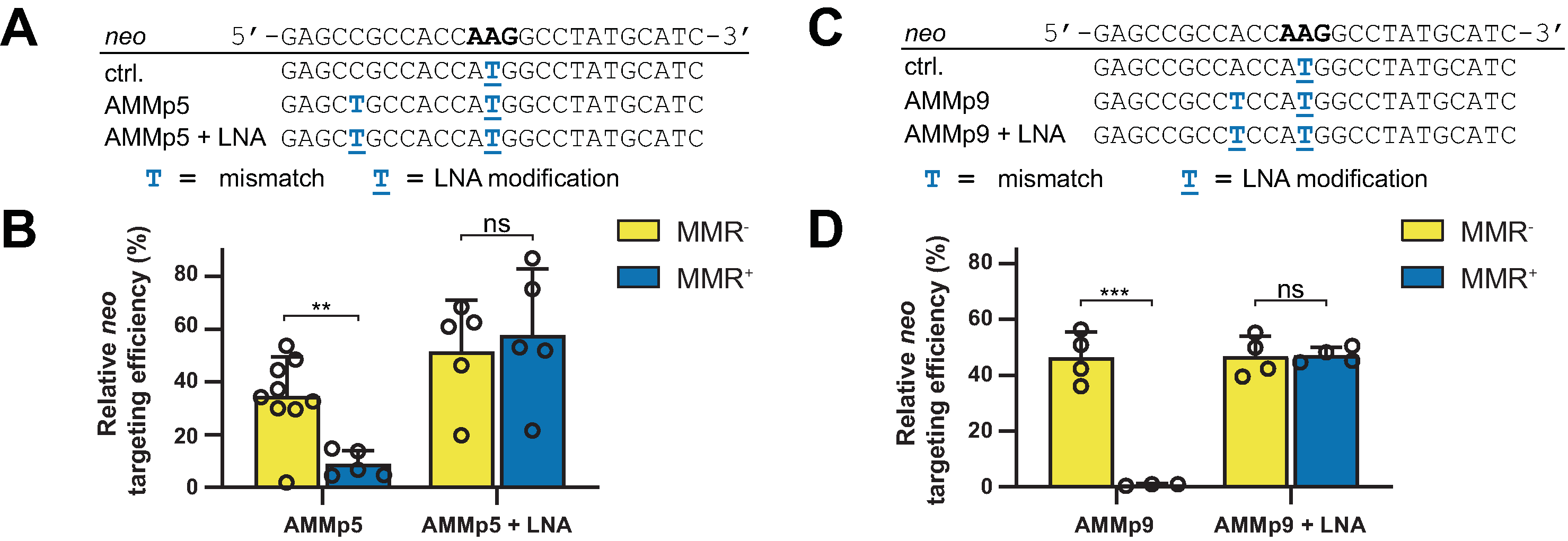

Supplement: S1 Fig — (A, B) Sequence (A) and relative neo targeting efficiency in MMR- and MMR+ cells (B) of AMMp5 LMOs with or without second LNA modification on p5. Significance was determined using a corrected multiple t-test. (C, D) Sequence (C) and relative neo targeting efficiency in MMR- and MMR+ cells (D) of AMMp9 LMOs with or without second LNA modification on p9. Bars indicate the mean with SD of at least five (B) or three (D) experiments. Blue capital characters indicate mismatches with respect to the reporter, underlined characters indicate LNA modifications. Significance was determined using a corrected multiple t-test. (TIF) [file pgen.1009041.s001.tif]

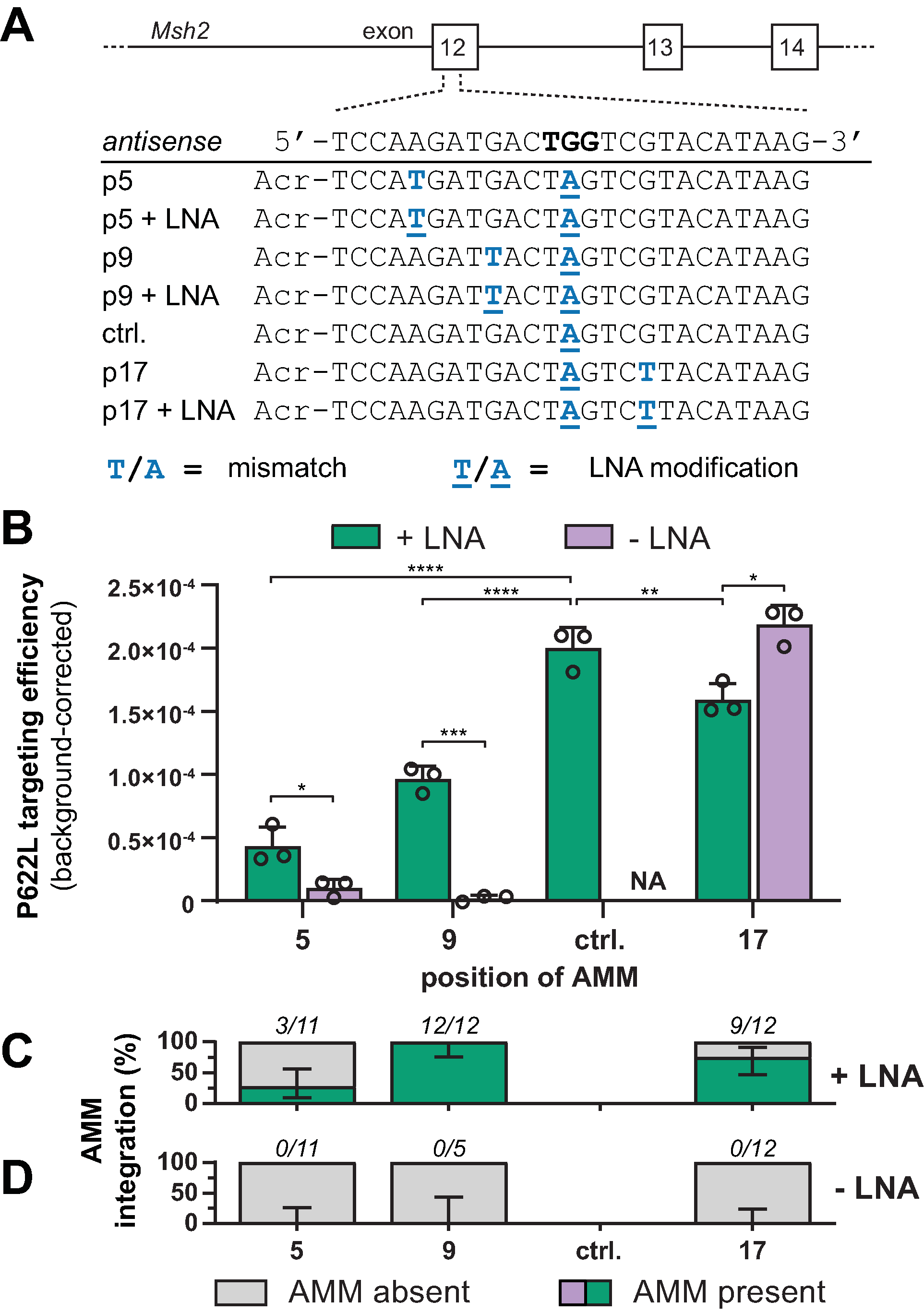

Supplement: S2 Fig — (A) Sequence of 5’-Acr modified antisense Msh2 P622L LMOs with single AMMs. Blue capital characters indicate mismatches with respect to Msh2 exon 12, underlined characters indicate LNA modifications. (B) Background-corrected targeting efficiencies of LMOs with a single AMM in Msh2+pur/Δ cells. LMOs with an LNA on p5, 9 or 17 (+LNA) were used to mimic targeting in MMR- cells. A non-specific LMO was used to determine the rate of spontaneous 6TGR background colony formation. NA indicates not applicable. Bars indicate the mean with SD of three experiments. Significance for comparing LMOs with different AMMs was determined using a corrected one-way ANOVA; significance for comparing LMOs with and without additional LNA modification was determined using a corrected multiple t-test. (C, D) Proportion of 6TGR colonies in which the indicated AMM was integrated after targeting with single AMM LMOs with additional LNA (+LNA, (C)) and without additional LNA (-LNA, (D)) as determined by Sanger sequencing. Error bars represent 95% confidence interval. (TIF) [file pgen.1009041.s002.tif]

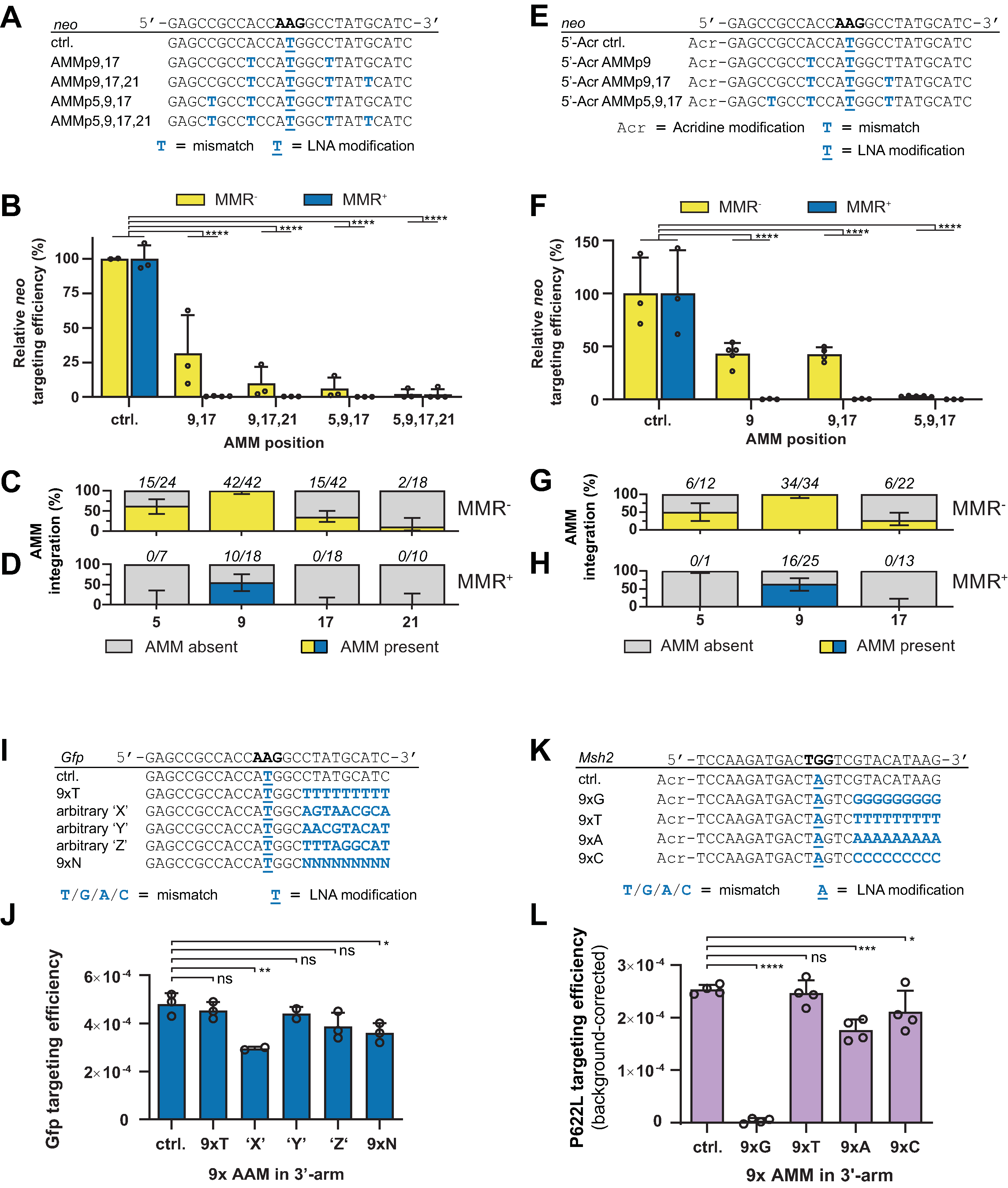

Supplement: S3 Fig — (A, B) Sequence (A) and relative neo targeting efficiency in MMR- and MMR+ cells (B) of LMOs with two, three or four AMMs. Blue capital characters indicate mismatches with respect to the reporter, underlined characters indicate LNA modifications. (C, D) Frequency of AMM integration at indicated positions after targeting with LMOs from (A) in MMR- (C) and MMR+ cells (D) as determined by Sanger sequencing. (E, F) Sequence (E) and relative neo targeting efficiency in MMR- and MMR+ cells (F) of 5’-Acr modified LMOs with one, two, or three AMMs. Bars indicate the mean with SD of at least three experiments. (G, H) Frequency of AMM integration at indicated positions after targeting with 5’-Acr LMOs from (E) in MMR- (G) and MMR+ cells (H) as determined by Sanger sequencing. Error bars (C, D, G, H) represent 95% confidence intervals. (I, J) LMOs with non-homologous 3’-arms (I) and targeting efficiency (L) for Gfp reporter in MMR+ cells. LMO 9xN represents a mix of LMOs in which the 3’-arm contains nine randomly introduced nucleotides. Bars indicate the mean with SD of two or three experiments. (K, L) 5’-Acr-modified antisense LMOs introducing the pathogenic P622L substitution in Msh2 containing mononucleotide tracts in the 3’-arm (K) and corrected targeting efficiencies (L). Bars indicate the mean with SD of four experiments. Significance was determined using a corrected two-way (B, F) or one-way ANOVA (J, L). (TIF) [file pgen.1009041.s003.tif]

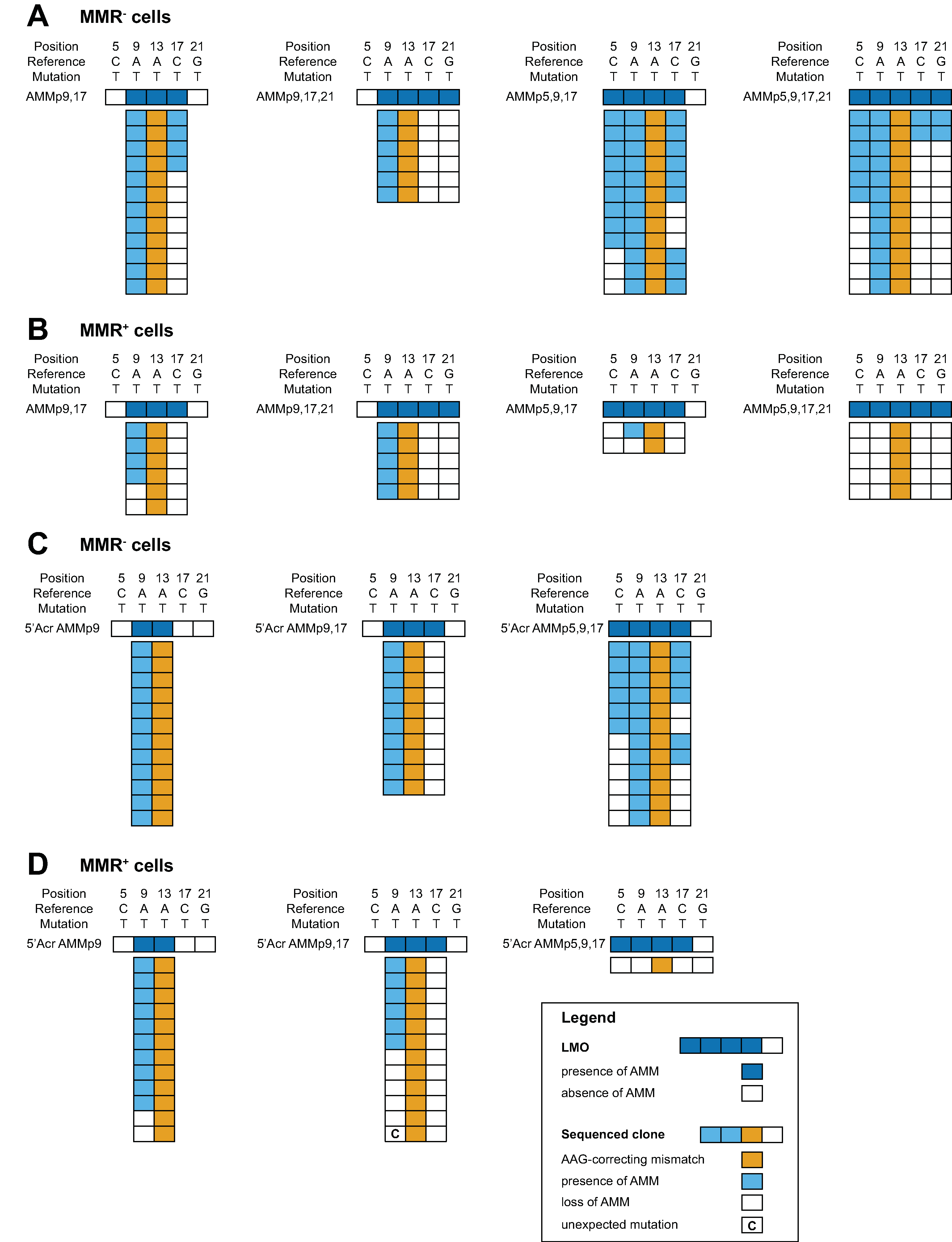

Supplement: S4 Fig — (A, B) Sequencing data from individual colonies (rows) modified by LMOs with multiple AMMs in MMR- (A) and MMR+ (B) cells. (C, D) Data from cells targeted with 5’-Acr modified LMOs with one or multiple AMMs in MMR- (C) and MMR+ (D) cells. Total integration frequencies per position are presented in S3 Fig. (TIF) [file pgen.1009041.s004.tif]

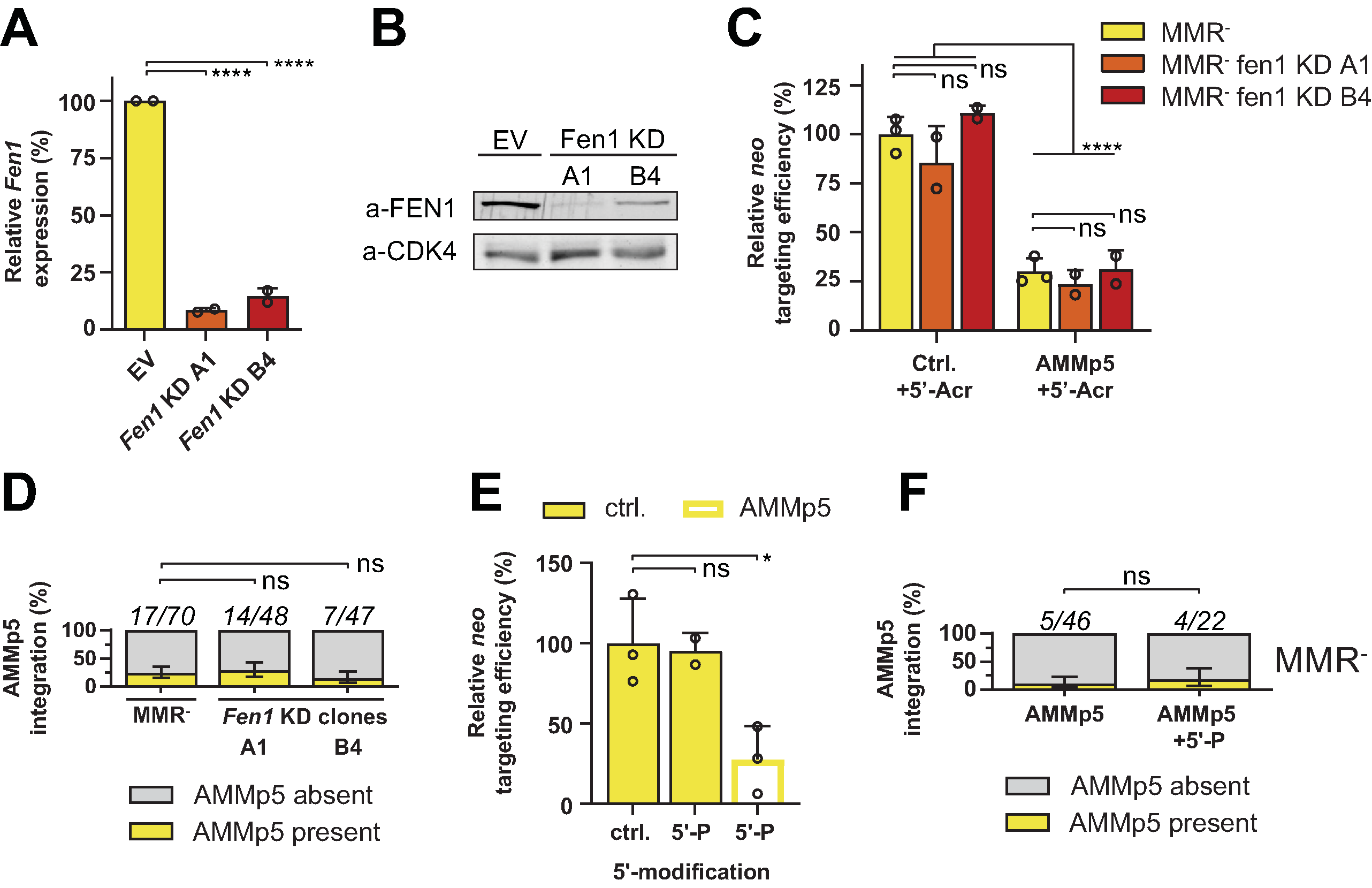

Supplement: S5 Fig — (A, B) Quantification of Fen1 gene expression by RT-qPCR (A) and FEN1 protein levels by western blot (B) in two independent stable Fen1 KD clones generated by lentiviral shRNA integration in MMR- mESCs with neo reporter. Fen1 expression was normalized against β-actin expression and data was obtained in two experiments with two technical replicates; bars indicate mean with SD. Significance was determined using a corrected one-way ANOVA. (C) Relative neo targeting efficiency using a 5’-Acr modified control and AMMp5 LMO in parental and Fen1 KD clones A1 and B4. Efficiency was normalized against efficiency obtained with the control LMO in the parental cell line. Bars indicate the mean with SD of at least two experiments. Significance was determined using a corrected two-way ANOVA. (D) Proportion of G418R colonies with integration of AMMp5 in MMR- Fen1 KD clones A1 and B4 after targeting with 5’-Acr modified AMMp5 LMO as determined by Sanger sequencing. (E) Relative neo targeting efficiency of 5’-phosphate modified LMOs in the presence and absence of AMMp5; data from at least two experiments. Significance was determined using a corrected one-way ANOVA. (F) Proportion of MMR- G418R cells in which AMMp5 was integrated. Error bars in (D) and (F) represent 95% confidence interval. (TIF) [file pgen.1009041.s005.tif]

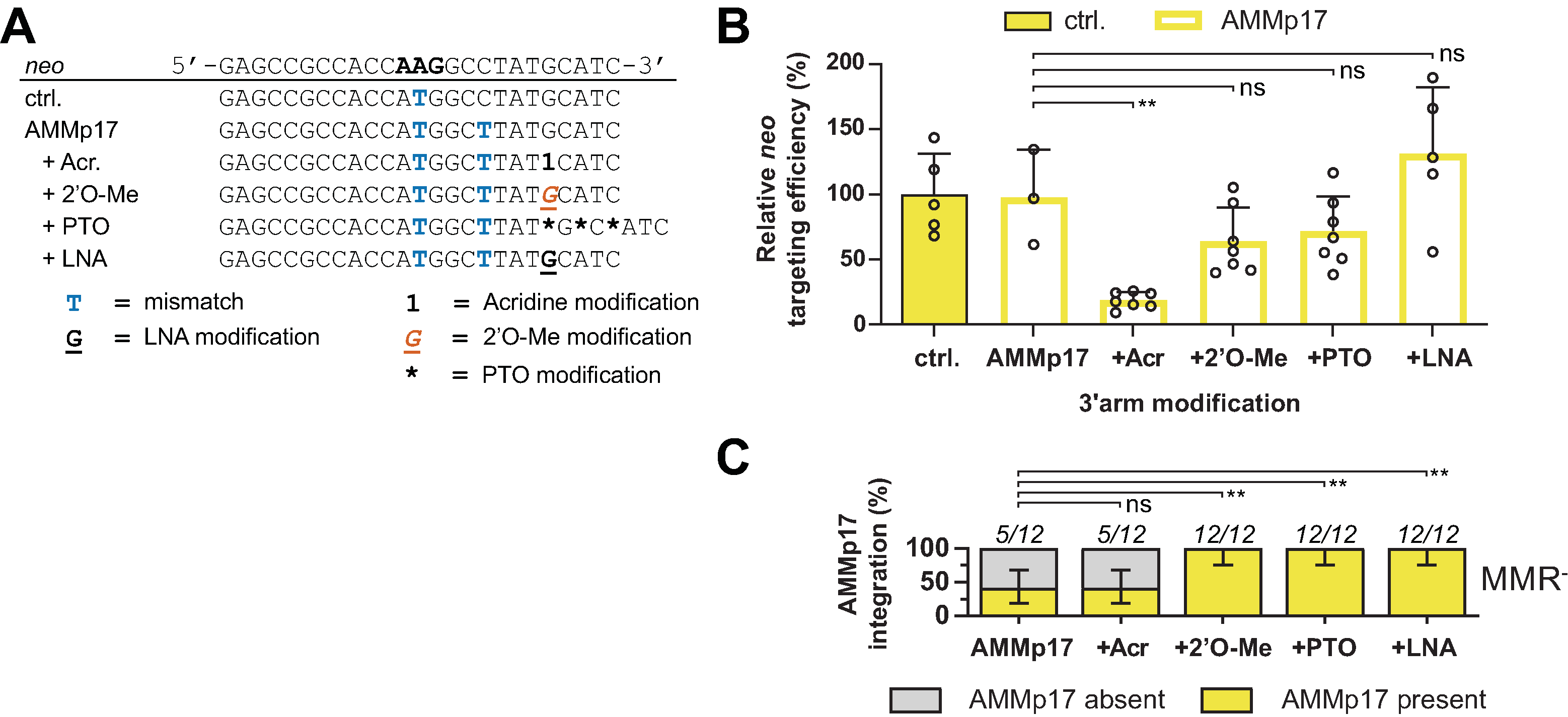

Supplement: S6 Fig — (A) Sequences of ssODNs (without LNA modification of the AAG-correcting central nucleotide) with AMMp17 and various 3’-arm modifications. Blue capital characters indicate mismatches with respect to the neo reporter, underlined characters indicate LNA modifications, ‘1’ indicates internal modification with 6-chloro-2-methoxyacridine, red underlined capital characters indicate modification with 2’O-Methyl nucleotides and asterisks indicate PTO-modified bonds. (B) Relative neo targeting efficiency in MMR- cells with AMMp17 containing ssODNs in combination with 3’-arm modifications. Bars indicate the mean and SD from at least three experiments. Significance was determined using a corrected one-way ANOVA. (C) Proportion of MMR- G418R colonies in which AMMp17 was integrated. Error bars represent 95% confidence interval. (TIF) [file pgen.1009041.s006.tif]

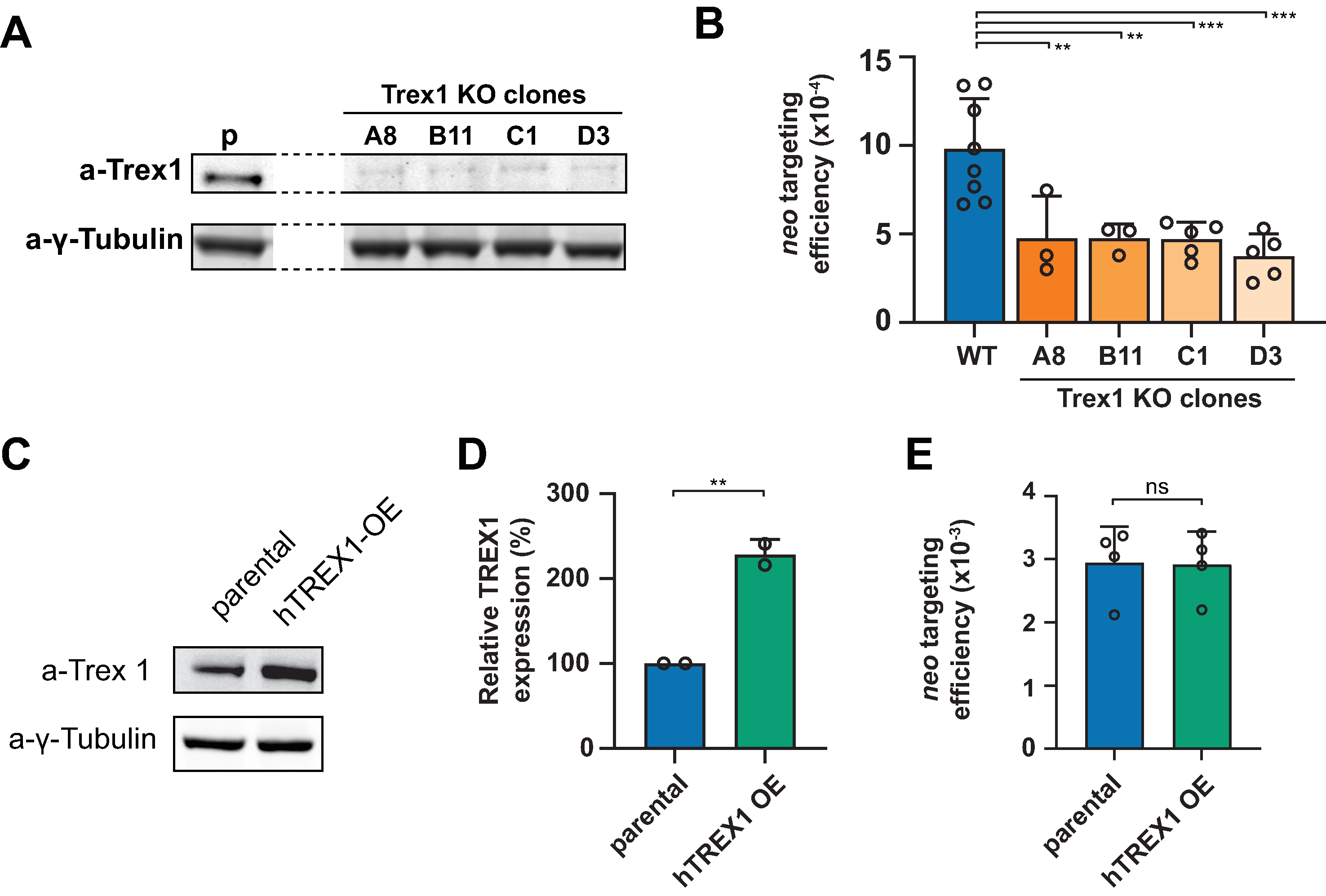

Supplement: S7 Fig — (A) Confirmation of CRISPR/Cas9 mediated knockout of TREX1 in MMR+ mESCs by western blot. (B) Efficiency of neo targeting in four TREX1 KO clones with 400 pmol 25 nt LMOs. Bars indicate the mean with SD of at least three experiments. Significance was determined using a corrected one-way ANOVA. (C, D) Validation (C) and quantification (D) of hTREX1 overexpression (OE) in MMR+ mESCs by western blot. Bars indicate mean and SD from two experiments. (E) Neo targeting efficiency with 5’-Acr modified LMO in MMR+ parental and TREX1 OE cells. Bars indicate mean and SD from four experiments. Significance was determined using a student’s t-test. (TIF) [file pgen.1009041.s007.tif]

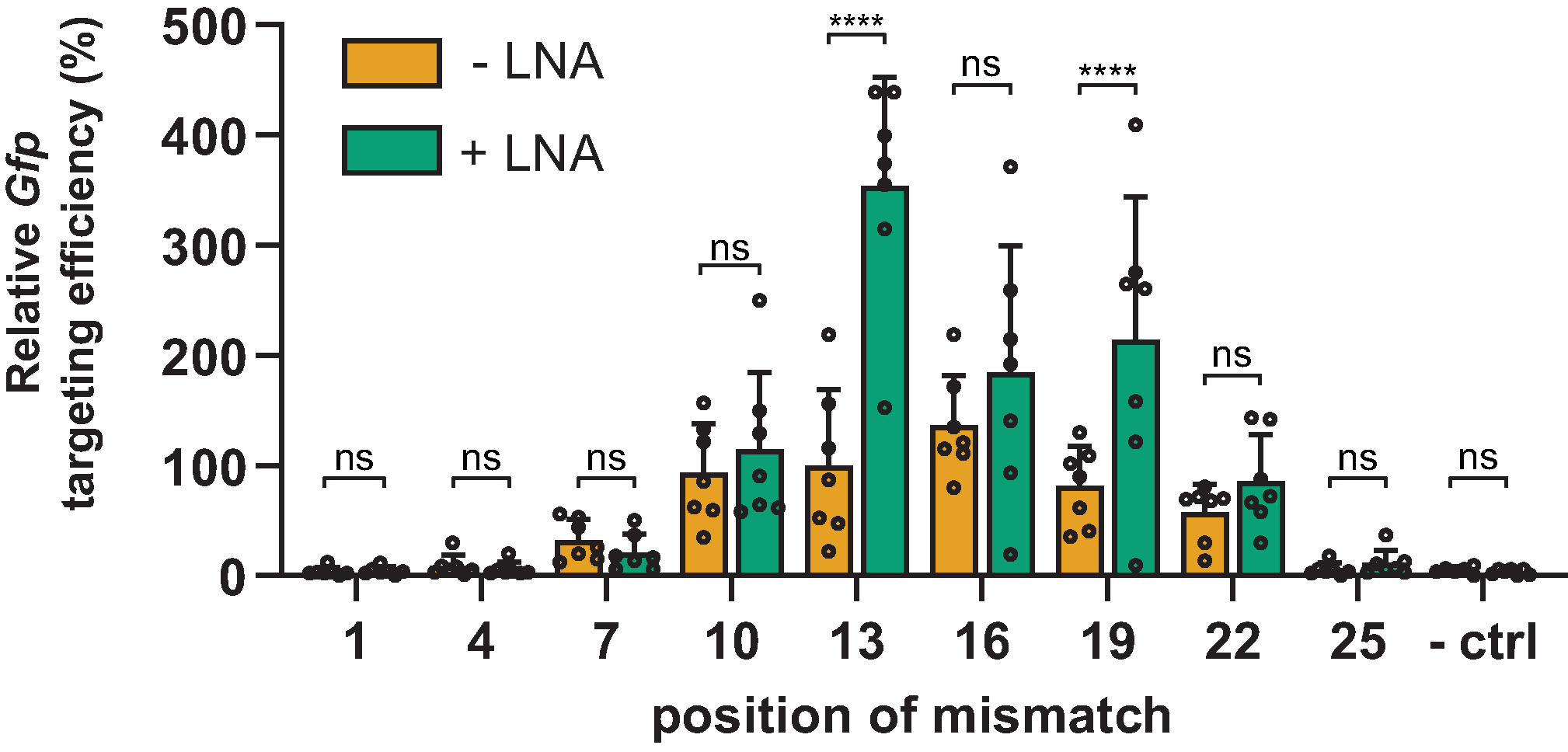

Supplement: S8 Fig — Relative Gfp targeting efficiency of ssODNs in presence and absence of LNA modification on the AAG-correcting mismatch in MMR- cells. Efficiency was normalized to ssODN with centrally positioned mismatch (p13) without LNA. Bars indicate mean with SD from seven experiments. Significance was determined using a corrected two-way ANOVA. (TIF) [file pgen.1009041.s008.tif]
