# Supplementary material for: Extensive trimming of short single-stranded DNA oligonucleotides during replication-coupled gene editing in mammalian cells
Source: PLoS Genet. 2020 Oct 29;16(10):e1009041. doi: 10.1371/journal.pgen.1009041 (PMC7595315; doi:10.1371/journal.pgen.1009041)
Supplement: Attachment — Submitted filename: ssODN.docx [file pgen.1009041.s010.docx]

The work titled “Extensive trimming of short single-stranded DNA oligonucleotides during replication coupled gene editing in mammalian cells” investigates the use of 25nt ssODNs to stimulate HR in dividing mouse ES cells. Different chromosomal reporters are used to determine gene editing in cells under selection and in one case using a GFP+ screen. Investigations are performed in MSH2(KO), FEN1(Kd), or TREX (KO) clones in order to provide mechanistic insight for what is perceived to be essentially an ssODN report to generate a more efficient repair molecule for enhanced editing via nucleotide modifications. Based largely on colony formation under selective conditions, and direct sequencing, interesting finding are reported including: i) the role of MMR in AMM in the 5’ arm yet not in the 3’ arm, ii) the action of an endonuclease in the 5’ homologous arm, and iii) the largely sequence independence of the 3’ arm presumably due to 3’ exonuclease activity prior to target annealing. A model is proposed to rationalize their findings and a rather modest enhancement in their oligo technology for gene editing is realized.

Overall, this manuscript is well written and the executed experiments are well controlled. Using largely indirect assays such as counting colonies under selection quite broad theories are proposed to explain the findings. Precise effectors/proteins involved in their model remain to be identified casting minor skepticism as to the proposed model, which, in itself, is sensible. The lack of direct data demonstrating 5’ endonucleolytic activity and or 3 prime degradation, especially with interpretations of enhancement of gene editing, appears reaching. However, overall, the paper represents an incremental increase in largely oligonucleotide design for modest enhancements in gene editing andprovides a thought provoking, yet largely unconfirmed, model that compiles the observed results. Overall, this paper is recommended for publication following a revision that adequately addresses the following specific concerns.

- The notion that 5’ arm AMM are not present in the target because of removal by endonucleolytic activity (when the 5’ ends are hypothesized to be protected from exonucleolytic activity by LNA or Acr) is interesting. This FEN1-independent mechanism is supported by internal PTO modifications in a position dependent manner in that if the PTO was at the AMM then incorporation was dramatically increased. This experiment appears to be only performed in MMR- cells - what is the result in normal mESCs? Can the authors speculate on which endonuclease may be involved and is process, and discuss potentially other non-endonucleolytic reasons for the observed result which in part, are largely indirect (not cleavage or evidence of this at the actual site).
- The concept of gene modification promoted by 3’arm degradation could certainly be interpreted simply as LNA modification of the mismatch stimulates incorporation independent of 3’ arm degradation. This alternate hypothesis is supported by no change in G419R colonies between AMMp17 and LNAp17 or LNAp25 (LNAp21 seemed to be position specific). Is it possible to provide direct evidence of 3’ degradation via HIRT ssODN isolation and polyacrylamide analysis? All the evidence is largely indirect and although the interpretation is sensible and well controlled, it’s still merely suggestive.
- The results of Fig. 5 and Fig. S8 are difficult to interpret based on the earlier data in the paper, including the endonucleolytic activity and exonucleolytic activity in the 5 arm and the notion that 3’ end processing (likely prior to annealing) enhances the AMM incorporation (editing).
- The results are largely indirect based on colony formation/counting, different oligos will have different target affinities (and modifications will also alter target interactions), altered stabilities, different off target promiscuities (and attractions), different inter and intra oligo associations, and many more hard to control concerns which may confound result interpretation – the authors should discuss these possibilities in relations to their conclusions and their proposed model.
- What was the transfection efficiency in these experiments and what is the ssODN copy # per cell?
- What was the rationale for choosing T as the mismatched nucleotide? Would other nucleotides alter the observed results?
- Do MSH2 cells have altered growth rates or hypermutability that may influence result interpretation as analyzed by colony formation. As MSH2 effects many aspects other than specifically MMR was MSH6 considered to decrease these concerns?
- Why in the absence of MMR were mismatches not incorporated in G418R colonies? Why in the presence of MMR was the p9 mutation incorporated in all cases?
