# Supplementary material for: Extensive trimming of short single-stranded DNA oligonucleotides during replication-coupled gene editing in mammalian cells
Source: PLoS Genet. 2020 Oct 29;16(10):e1009041. doi: 10.1371/journal.pgen.1009041 (PMC7595315; doi:10.1371/journal.pgen.1009041)
Supplement: Attachment — Submitted filename: Point-by-point reply_final.docx [file pgen.1009041.s011.docx]

**Manuscript PGENETICS-D-20-00005**

**Point-by point reply to Reviewer Comments and Questions**

All three reviewers have positively evaluated our manuscript, but also made some important comments. We thank the reviewers for their efforts and constructive suggestions that greatly helped us to improve our manuscript. We did our best to adequately address each of the points raised by the reviewers, in some cases supported by novel experiments, and hope these improvements make our manuscript eligible for publication in PLoS Genetics.

***Reviewer #1:***

*“The study is well written, experiments are planned well, and the results are of significant interest in the gene editing/DNA repair community. However, the value of the LMO technology is not immediately clear because frequencies of gene editing are not evident, and the risk of off-target DNA modifications is not taken into consideration.”*

It is true that the frequency of gene editing by LMOs is modest, reaching efficiencies of 10^-3^. However, the technique is highly precise which makes it particularly useful to study variants of uncertain clinical significance (VUS) that confer a selectable phenotype, as we refer to in the Introduction (PNAS 2016;113:4128).

**Modification: we now discuss the applicability of LMO technology in interrogating VUSs of DNA mismatch repair genes that underlie the cancer predisposition Lynch syndrome more extensively (P15L321-329). Also implications from our results for possible off-target modifications are being discussed (P13L278-284).**

*Major points:*

*“- In the Results, it will be valuable to provide what is the actual frequency of gene editing in the mESC using the LMOs for the correction of the neomycin reporter gene.”*

**Modification: we have included the *neo* reporter gene correction efficiency for our standard control LMO of 25 nt in the results section (P5L99-100).**

*“Is this frequency sufficiently high for editing DNA in cases in which there is no change in phenotype after the gene correction?”*

The frequencies we now obtain are already sufficiently high to rapidly purify modified cell clones using a single point-mutation-specific PCR protocol, as we used previously (Mutat Res 2011;715:52). Nevertheless, we will continue our efforts to increase the performance and applicability of replication-coupled gene editing in mammalian cells.

*“How does LMO technology with no break in the target locus compare to gene editing following DNA break induction?”*

Our LMO-based gene targeting approach seems currently less efficient than CRISRPR-based technologies, although CRISPR/Cas sometimes completely fails. *E.g.*, we showed that oligonucleotide-mediated repair of a CRISPR-induced DNA double strand break in the same stably integrated GFP reporter in mESCs yielded an efficiency of up to 40%, albeit concomitant PAM disruption was required to prevent re-cutting (NAR 2018;46:2945). While LMO technology is clearly less efficient, the lack of undesired on-target modifications enables direct phenotypic assessment of introduced variants. Such an approach is less feasible using DSB-inducing techniques as DNA repair through non-homologous end-joining often results in unforeseen insertion/deletion mutants.

**Modification: we have extended the Discussion section with an additional paragraph in which we compare and discuss the efficiencies obtained from our LMO-based technology with our results obtained for CRISPR-based technologies from Harmsen *et al.* (NAR 2018;46:2945) (P15L316-321).**

*“Considering the results of this study, it will be important to clearly discuss what can be most significant applications of the LMO technology.”*

**Modification: in addition to the comparison of LMO- and CRISPR-based technologies we also discuss present and potential future applications of LMO technology related to gene variant characterization (P14L321-329).**

*“-Showing Western blot data for FEN1 KD would be a much stronger result than RT-PCR. RT-PCR does not provide a measure of the protein level in the cells. Thus, in this study, it cannot be concluded that FEN1 has no effect on gene editing by LMOs in mESCs.”*

We agree that Western blot data are imperative to support our conclusion that FEN1 knockdown does not affect introduction of 5’-located mutations.

**Modification: we have included Western blot data in supplemental Figure S5, updated the methods and materials (P18L398-401) and have adjusted the supporting information caption accordingly (P29L626).**

*“-If most of the 3’-end of the LMOs can be of any sequence (NNNNNN) and only ~ 8nt of the 25mer LMOs are integrated in the DNA at the site of gene editing, the LMOs can likely recognize multiple targets, in part like miRNAs do. Did the authors examined whether the LMOs are integrated at other sites? Does this small size of complementarity with the target sequence represent a risk for off-target editing? It would be valuable if the authors could also discuss the value of LMO-driven gene editing with respect to its risk of off-target modifications.”*

This is a very interesting point. We realize that both, the higher frequencies of LMO targeting we now reach and our finding that only a small part of LMOs become integrated into the genome, make possible off-target integrations a greater concern than before. This topic has attracted our attention and will be investigated in more detail in the future.

**Modification: we have added an additional paragraph to the discussion-section in which we discuss the relevance of potential LMO off-target edits and make a recommendation to limit potential off-target integrations (P13L278-284).**

*Other comments:*

*“-The authors write on page 5: <>. It would be helpful to explain what does “normalized efficiencies” mean in this context, and exactly what was done to normalize the data.”*

**Modification: we have clarified our data normalization procedure in the Methods section (P16L358-362).**

*“-In Figure 1C and D (and other Suppl figures), it is not clear what is the meaning of the confidence interval. Were the samples sequenced multiple times, how many times? How many colonies were sequenced from each individual transfection experiment? For example, 24/24 does it meant that there were 24 G418 colonies among the 3 repeats (shown in Fig. 1B) and all of them were sequenced, how many from each of the 3 transfections?”*

The presented sequencing data is based on the sequencing outcome of individually picked colonies that were sequenced once. Colonies were picked in one or more replicate experiments. *E.g.*, 5/46 indicates that in total 46 unique G418^R^ colonies were sequenced, of which 5 yielded the AMM of interest. Since our AMM sequencing data provided only two possible results, AMM integration or not, sequencing results were summarized and presented as proportions. As the outcome of our observational sampling provides only an estimation for the true proportion within the overall population, we used the 95% confidence interval (calculated by Wilson/Brown method) to quantify and show this uncertainty.

**Modification: we have made two modifications to the Methods section. First, we clarified that colonies were picked from one or more replicate experiments P17L372). Second, we indicated that the 95% confidence interval was used to quantify the uncertainty of the calculated proportions based on our sequencing data (P19L408-409).**

*“-Did the authors examine whether random sequence, which is not a homonucleotide run, in the 3’-arm also has no impact of gene editing like multiple As, Cs, and Ts?”*

This is an interesting idea. We have performed several new experiments with randomly chosen 3’-arm sequences and essentially found none of them to impact effective targeting.

**Modification: We present these novel data in supplementary figure S3I, J and adjusted the figure caption accordingly (P29L614-617).**

*“-Should correct in Figure S7D Fen1 to Trex1 in the legend of the Y axis.”*

**Modification: we have corrected the legend of the y-axis in Fig S7D.**

***Reviewer #2:***

*“Overall, this manuscript is well written and the executed experiments are well controlled. Using largely indirect assays such as counting colonies under selection quite broad theories are proposed to explain the findings. Precise effectors/proteins involved in their model remain to be identified casting minor skepticism as to the proposed model, which, in itself, is sensible. The lack of direct data demonstrating 5’ endonucleolytic activity and or 3 prime degradation, especially with interpretations of enhancement of gene editing, appears reaching. However, overall, the paper represents an incremental increase in largely oligonucleotide design for modest enhancements in gene editing and provides a thought provoking, yet largely unconfirmed, model that compiles the observed results.*

We do agree with the reviewer that our conclusions and the model we present are based on indirect evidence, *i.e.*, on colony counts and sequencing of modified alleles. However, what counts in our opinion is the fate of the ssODN that ultimately ends up in the chromosome to effectuate the desired modification. Given the relatively modest frequency of successful targeting events, it is uncertain whether biochemical information on the fate of the total oligonucleotide population that has entered cells, such as degradation, location etc., is representative for the ‘winning oligonucleotide”.

**Modification: we made a note of this concern of the reviewer and explain why we focus on successful targeting events (P12L267-277).**

*Overall, this paper is recommended for publication following a revision that adequately addresses the following specific concerns.”*

*“The notion that 5’ arm AMM are not present in the target because of removal by endonucleolytic activity (when the 5’ ends are hypothesized to be protected from exonucleolytic activity by LNA or Acr) is interesting. This FEN1-independent mechanism is supported by internal PTO modifications in a position dependent manner in that if the PTO was at the AMM then incorporation was dramatically increased. This experiment appears to be only performed in MMR- cells - what is the result in normal mESCs?”*

We show that in normal mESCs AMMs in the 5’-arm are subject to MMR and hence not present in targeted cells (Fig. 1B, C). This means that MMR surveillance precedes and likely replaces 5’-arm degradation. The goal of the experiment in which we include internal PTO modification was to gain insight in the process of 5’-arm degradation. As internal PTOs may still allow MMR-dependent removal of 5’-arm sequences, this experiment has only been done in MMR^-^ mESCs. In this way, we avoid confounding effects of MMR.

*“Can the authors speculate on which endonuclease may be involved and is process, and discuss potentially other non-endonucleolytic reasons for the observed result which in part, are largely indirect (not cleavage or evidence of this at the actual site).”*

While 5’-end modifications probably enhance targeting by providing protection from exonucleases, we observe that AMMs in the 5’-arm strongly suppress targeting, especially in MMR^+^ cells. Based on our observations we strongly believe that an intact 5’-arm is required for successful annealing of the LMO to its target site. Hence, the 5’-arm degradation most likely takes place after annealing.

Based on our current observations we do not envisage a major role for non-endonucleolytic processes relevant for 5’-arm degradation after annealing. Unfortunately we haven’t been able to conclusively determine the role of FEN1 in endonucleolytic degradation of the 5’-arm. As FEN1 is regulated through various post-translational modifications and has multiple interaction partners (reviewed by Balakrishnan and Bambara 2013, Annu Rev Biochem 2013;82:119-3), it’s not straightforward to assess its role in detail.

Besides FEN1 there are a couple of structure-specific endonucleases (SSEs) (reviewed by Dehé and Gaillard 2017, Nat Rev Mol Cell Biol;18(5):315-330) that could affect 5’-arm degradation. For example, XPG is known to be activated by a 5’-flap during nucleotide excision repair and incises the damaged strand 3’ of the lesion. Moreover, as annealing LMOs may influence local replication fork dynamics, factors that have an important role in replication fork restart and mitigation of replication stress, *e.g.*, MUS81, ZRANB3 and FAN1, might influence 5’-arm degradation as well. Other candidates include XPF-ERCC1 and SLX1, which have been mostly studied for their role in interstrand-crosslink repair. Less likely candidates are active outside S-phase, for example GEN1, an endonuclease that depends on nuclear envelope breakdown during mitosis to access chromosomal DNA. Although we cannot fully exclude them, we also consider sequence- or modification-specific endonucleases such as APE1 and APE2 less likely candidates for the observed loss of 5’-arm sequence information.

**Modification: we have highlighted in the Discussion section that FEN1 is an important candidate for 5’-arm degradation, even though we haven’t been able to conclusively determine its role. Moreover we propose that other SSEs could influence 5’-arm degradation (P14L300-305).**

*“The concept of gene modification promoted by 3’arm degradation could certainly be interpreted simply as LNA modification of the mismatch stimulates incorporation independent of 3’ arm degradation. This alternate hypothesis is supported by no change in G419R colonies between AMMp17 and LNAp17 or LNAp25 (LNAp21 seemed to be position specific).”*

Interpretation of the 3’-arm degradation experiments is indeed not straightforward. We observed that LNA modification at either p17 or p21 enhanced the integration rate of AMMp17 from ~20% to ~70% in MMR^-^ cells, suggesting that LNA at p17 and p21 are at least partially protective for 3’-5’ degradation. Somewhat puzzling, suppression of 3’-arm degradation by LNA did reduce targeting efficiency of LNAp21 but not LNAp17. Following the reviewer’s interpretation this may indicate that 3’-arm degradation can happen but is not a requirement for effective targeting. However, one may also argue that 3’-arm degradation up to position 17 is sufficient for optimal targeting whereas degradation limited to a few nucleotides 3’ of position 21 is not. How then explain the result of LNAp25 that performs as efficient as the control LMO? Perhaps the reduced incorporation of the AMM at position 17 indicates that 3’-arm degradation was not effectively suppressed. However, we realize that the number of analyzed colonies was small and conclusions should be drawn with caution. Taken together with our finding that loss of 3’-5’ exonuclease TREX1 led to reduced targeting efficiencies, we propose the results in Fig. 4 indicate that 3’-arm degradation is likely to be beneficial for targeting.

**Modification: While we believe our data provide strong evidence for 3’-arm degradation (most strikingly visible in Fig. 2), we have in the Discussion section more extensively described why we think that 3’-arm degradation may be beneficial for effective targeting (P12L259-264).**

*“Is it possible to provide direct evidence of 3’ degradation via HIRT ssODN isolation and polyacrylamide analysis? All the evidence is largely indirect and although the interpretation is sensible and well controlled, it’s still merely suggestive.”*

We do agree with the reviewer that our conclusions and the model we present are based on indirect evidence, *i.e.*, on colony counts and sequencing of modified alleles. The suggestion of the reviewer to test whether ssODNs entering the cell are subject to 3’ degradation is certainly interesting. However, what counts in our opinion is the fate of ssODNs that ultimately end up in the chromosome to effectuate the desired modification. Given the relatively modest frequency of successful targeting events, it is uncertain whether biochemical information on the fate of the total oligonucleotide population that has entered cells, such as degradation, location etc., is representative for the ‘winning oligonucleotide”.

*“The results of Fig. 5 and Fig. S8 are difficult to interpret based on the earlier data in the paper, including the endonucleolytic activity and exonucleolytic activity in the 5 arm and the notion that 3’ end processing (likely prior to annealing) enhances the AMM incorporation (editing).”*

We agree with the reviewer that the results in Fig. 5 and Fig S8 may be difficult to interpret. However, we believe they very well represent the outcome of the interplay of the 5’- and 3’-arm degradation processes we identified. Endonucleolytic degradation of 7-8 nt of the 5’-arm *after* annealing of an LMO to its target site explains why LMOs with an AAG-correcting mutation repositioned towards the 5’-arm are largely ineffective. So, P1, 4, 7 hardly work because the 5’-arm including the AAG-correcting mismatch is completely removed, while p10 starts to give a reasonable frequency. Exonucleolytic degradation of the 3’-arm comprises 9 nt or proceeds until the exonuclease encounters the LNA-modified AAG-correcting mutation. We envisage that for p13 three nucleotides of the 3’-arm are left, while for p16, 19 and 22 only 1-2 nt are left, which may destabilize the heteroduplex around the correcting mismatch, explaining why these LMOs perform slightly less effective than p13. So, it appears that the central position is mostly likely to ‘survive’ the degradation processes most optimally and therefore yields the highest efficiencies.

**Modification:** **we have clarified in the Results-section that the complex interplay between 5’- and 3’- arm degradation leads to an outcome in which a centrally positioned mismatch is optimal due to its likelihood to remain unaffected by both degradation processes (P11L235-237).**

*“The results are largely indirect based on colony formation/counting, different oligos will have different target affinities (and modifications will also alter target interactions), altered stabilities, different off target promiscuities (and attractions), different inter and intra oligo associations, and many more hard to control concerns which may confound result interpretation – the authors should discuss these possibilities in relations to their conclusions and their proposed model.”*

Our conclusions and working model were indeed based on the combination of sequencing data and targeting efficiencies. Given the modest efficacy of LMO targeting, we feel this is the only way to divulge the fate of the oligonucleotides that have actually effectuated the gene modification (the ‘winning oligonucleotide’). The vast majority of oligonucleotides does not participate in the actual gene modification and their fate may be different from the successful LMOs. Although most data was obtained by evaluating the efficiencies of neo/Gfp targetings using highly similar oligonucleotides, we realize the presence of AMMs and chemical modifications may induce unexpected variations in oligonucleotide behavior that may influence our results. On the other hand we have validated our most striking findings at the *Msh2* locus.

**Modification: we have included an additional paragraph in the Discussion-section in which we explain our choice for focusing at the ‘winning oligonucleotide’ and discuss uncertainties associated with unforeseen consequences of different oligonucleotide designs (P12L267-277).**

*“What was the transfection efficiency in these experiments and what is the ssODN copy # per cell?”*

Using SiQuest (Mirus), transfection of a 25 nt LMO with a covalently linked 5’ fluorescent dye (ATTO488) led to detectable fluorescence in ~70-75% of mESCs as analyzed by flow cytometry.

A rough estimation of the LMO copy number per cell after transfection could be made, assuming that all LMO molecules in the transfection medium successfully form transfection particles that precipitate on the surface of a single 6-well with ~1.2 x10^6^ cells that occupy ~75% of the surface of a 6-well at day of transfection.

Then transfection of 3 ug of 25 nt LMOs translates to (3 ug LMO * 0.75 cell surface occupation / 1.2x10^6^ cells =) 1,9 picogram = 246 x 10^-18^ mol = ~10^8^ LMO molecules per cell.

However, we do not take into account the transfection efficiency per cell, intracellular degradation or cellular division. Moreover, we observed endosomal entrapment of LMOs and therefore it’s hard to estimate how many LMO molecules reach the cell nucleus and are available for replication-coupled gene editing.

Given this high level of uncertainty we choose not to include this estimation in the manuscript.

*“What was the rationale for choosing T as the mismatched nucleotide? Would other nucleotides alter the observed results?”*

The AAG -> ATG correction requires a T but the choice for T for all AMMs was arbitrary; we could have chosen one of the other nucleotides as well. For single mismatches we do not a priori expect a different outcome if we would use a C, G or A for the AMMs, but we cannot fully exclude this.

**Modification: we have added this point to the additional paragraph in the Discussion-section in which we discuss uncertainties associated with unforeseen consequences of different oligonucleotide designs (P13L272-275).**

*“Do MSH2 cells have altered growth rates or hypermutability that may influence result interpretation as analyzed by colony formation. As MSH2 effects many aspects other than specifically MMR was MSH6 considered to decrease these concerns?”*

“We specifically choose *Msh2^-/-^* mESCs to study LMO degradation in absence of MMR since loss of MSH2 results in complete abrogation of the MMR pathway. Previously we described that replication-coupled gene editing is about 5-fold less efficient in *Msh6^-/-^* mESCs in comparison to *Msh2^-/-^* mESCs, likely due to suppression of targeting via activation of MMR by MSH2/MSH3 (Aarts et al. 2006, NAR;34(21):e147). So, in *Msh6^-/-^* cells certain mismatches may be subject to MMR and others not, which would create an unacceptable variation. Although MMR deficient cells are known to have a mutator phenotype, we haven’t observed an increased rate of spontaneous G418^R^ colony formation in *Msh2^-/-^* mESCs in our stringent neo targeting assays. Hence, result interpretation is not affected by hypermutability of *Msh2^-/-^* mESCs. In our experiments we observed that *Msh2^-/-^* mESCs had a slightly reduced proliferation speed, which may explain the 50% reduced targeting efficiency we observed for control LMOs in comparison to *Msh2^+/+^* mESCs. To enable fair comparison of targeting efficiencies between MMR^+^ and MMR^-^ cells we therefore normalized targeting efficiencies to the control LMO per cell line.

**Modification: we have specified our choice to use *Msh2*^-/-^ cells to abrogate MMR in the Results section (P5L100-101) and clarified the data normalization in the Methods section (P16L358-362).**

*“Why in the absence of MMR were mismatches not incorporated in G418R colonies? Why in the presence of MMR was the p9 mutation incorporated in all cases?”*

We interpret the absence of AMM incorporation in MMR^-^ cells as a direct readout for LMO degradation during the targeting. Comparing AMM incorporation in MMR^+^ and MMR^-^ cells enabled us to conclude that 3’-arm degradation takes place before and 5’-arm degradation after LMO-target site hybridization. As discussed in the results section, AMMp9 is indeed always incorporated in MMR^+^ cells but this is actually a very rare event as only very few G418^R^ colonies appeared. This is because in most targeting events AMMp9 activates MMR, which results in excision of both AMMp9 and the central neo correcting mutation. Apparently, in very few cells AMMp9 failed to activate MMR giving rise to a G418^R^ colony containing the AMMp9.

**Modification: We have clarified our interpretation for the observed introduction of AMMp9 in MMR^+^ mESCs in the Results (P6L121-122) and Discussion section (P12L247-250).**

***Reviewer #3:***

*“The figures are well drawn, and as in previous publications from this lab, having the sequences of the oligos in each figure, with designations of which ones contain LNAs and/or create mismatches, is very helpful. Designations in the figures of whether sequencing results are from MMR+ or MMR- hosts, while in the legends, would also help.”*

**Modification:** **we have added MMR^+^ and MMR^-^ labels to the sequencing data in all main and supplemental figures.**

*Minor points*

*“1. Why is the AMMp9 position one that is taken up by the colonies to such a high degree? On might argue that access is limited by the processivity of an exonuclease to that position, but the model favored by the authors is one of endonucleolytic processing of the 5’ arm. Might its proximity to the central LNA play a role in protection from degradation?”*

Based on our experimental data in which we used internal PTO modifications in the 5’-arm we conclude that 5’-arm degradation mainly takes place through endonucleolytic cleavage at p5-7. In addition we think that central mismatch p13 (conferring the AAG->ATG correction) can only be stably integrated if at least some base pairing nucleotides remain present at its 5’-side; thus, degradation of the 5’-arm beyond p8 may lead to destabilization of annealed LMOs, prohibiting gene correction. Together these mechanisms explain why AMMp9 is commonly co-integrated with the AAG correcting central mismatch in MMR^-^ cells. While sequencing data shows AMMp9 incorporation in MMR^+^ cells in all colonies, we would like to stress that this data represents only a very limited number of cells. In general we observe that AMMp9 leads to a strong reduction in targeting efficiency, indicating that MMR recognizes the AMMp9-induced mismatch and activates nucleases to destabilize or co-excise the central p13 mismatch. The rare colonies that did arise after targeting with LMO AMMp9 likely represent a very rare fraction of cells in which MMR was not properly activated.

**Modification: we have clarified our interpretation for the observed introduction of AMMp9 in MMR^+^ mESCs in the Results section (P6L121-122). Additionally, we briefly propose in the Discussion section that 5’-arm loss beyond p8 may destabilize annealed LMOs (P12L249-250).**

*“2. Line 106 “we found that MMR aggravated the suppressive effect of 5’-arm AMMs at p5”….
This sentence is a little unclear. May I suggest “…we found that the presence of MMR exacerbated the suppressive effect of 5’-arm AMMs at p5….”*

**Modification: Thank you for this suggestion; we have changed line 106 (P5L109).**

*“Also, perhaps the authors could discuss a mechanism for this observation. One might think that MMR activity on the AMM results in collateral damage of removing the LNA-containing targeting base, but wouldn’t that base be resistant to nucleases involved in MMR?”*

It is possible that the single LNA at the targeting base provides protection against MMR-induced exonuclease activity. After all, for AMMp5 LMOs MMR reduced the *neo* correction efficiency, but not to zero (compare targeting efficiency of AMMp5 LMO in MMR^+^ versus MMR^-^ cells in Fig. 1B). On the other hand, the MMR-dependent reduction we do see can be explained by MMR-dependent extensive shortening of the 5’-arm including the targeting base, either because LNA protection is not 100% or because shortening weakens the annealing of the LMO which results in formation and removal of a flap by an endonuclease.

**Modification: we have included this reasoning in the Discussion section (P12L247-250).**

*“3. Line 256 “Nevertheless, also in E. coli ssODNs showed a pattern of incomplete incorporation“
Change to: Nevertheless, E. coli ssODNs also showed a pattern of incomplete incorporation”*

**Modification: we have made this change to line 256 to improve readability (P13L289).**

*“4. Line 166-167 “Also the targeting-suppressive effect of AMMp5 in MMR- and even more in MMR+ cells was not affected by LNA at p1 or 5’-Acr modification of the LMO.”
It would help the reader to tell them to compare the y axes of Fig 3B and C.”*

Thank you for this suggestion. Indeed, while 5’-end modification by LNA p1 or 5’-Acr increases targeting efficiencies in general (Fig. 3B), in all cases AMMp5 reduced targeting efficiencies. This is clear from comparison of Fig. 3B and 3C, provided the reader realizes that in C the targeting efficiencies are reduced to about 40% of those in B.

**Modification: we have included a notification to indicate the y-axis dimension difference in Fig. 3B and C, stressing that in all cases, AMMp5 reduced targeting efficiencies ~2.5-fold (P8L171).**
